# Prevalence of minor physical anomalies in children with autism spectrum disorder reporting to a tertiary care hospital Lahore-Pakistan

**DOI:** 10.12669/pjms.38.7.6639

**Published:** 2022

**Authors:** Ansa Rabia, Saqib Mahmood, Shazia Maqbool

**Affiliations:** 1 Ansa Rabia, MPhil. University of Health Sciences, Lahore. Department of Anatomy, CMH Lahore Medical College & Institute of Dentistry, Lahore-Pakistan. National University of Medical Sciences, Rawalpindi-Pakistan. University of Health Sciences, Lahore, Pakistan; 2 Saqib Mahmood, PhD. University of Health Sciences, Lahore, Pakistan; 3 Shazia Maqbool, FRCPCH. Department of Developmental-Behavioral Pediatrics, University of Child Health Sciences & The Children’s Hospital, Lahore-Pakistan

**Keywords:** Autism spectrum disorder, dysmorphic, minor physical anomalies

## Abstract

**Objective:**

To assess the frequency and type of minor physical anomalies (MPAs) in subjects with autism spectrum disorder (ASD).

**Methods:**

This descriptive cross-sectional study was conducted from September, 2016 to October, 2020. Using purposive sampling technique, 147 subjects with ASD were recruited from Children’s Hospital and Institute of Child Health (CH & ICH) Lahore, with a confirmed clinical diagnosis by developmental pediatrician, using Diagnostic and Statistical Manual of Mental Disorders (DSM-V). For morphology assessment, 12 body regions of ASD subjects were examined using Autism Dysmorphology Measure (ADM) manual after taking informed consent. Physical measurements (height, weight, head circumference, ear length, philtrum, hand, finger and foot length) were also taken and were compared with the available standard charts.

**Results:**

A total of 381 dysmorphologies were identified in 131 (89.1%) ASD subjects whereas 16 subjects had no dysmorphology at all. Microcephaly was exhibited by 14 (9.5%) subjects, out of which 13 had variable number of dysmorphologies while one had no dysmorphology in other body regions. Out of 131 subjects exhibiting dysmorphologies, there were 108 male and 23 female subjects, with a M:F ratio 4.7:1 whereas microcephaly was observed in 12 male and two female subjects, with a M:F ratio 6:1. The highest number of dysmorphic features were noted in the ears, followed by feet and hair growth pattern.

**Conclusions:**

MPAs associated with ASD are frequently found in, but are clearly not limited to, the head or facial region.

## INTRODUCTION

Autism Spectrum disorder (ASD) is an early childhood onset, complex neurodevelopmental disorder. It is a life-long condition of problem in communication and behavior. According to the Centers for Disease Control and Prevention (CDC), 1% of the world population has ASD.^1^ Males are more likely than females to be affected with an approximate prevalence ratio of 4.4:1.^2^ In Asia an overall prevalence of ASD has been reported to be 0.36% whereas it is 0.31% in South Asia.^3^ Affected children have serious developmental disadvantages in terms of schooling and social inclusion preventing them from reaching their productive potential in society as adults.^4^ There is promising awareness regarding ASD in health professionals from Pakistan,^5^ however, individualized educational curriculum for these children needs to be provided.^6^

In subjects with ASD, high incidence of dysmorphic features in various body regions has been reported.^7^ Minor Physical Anomalies (MPAs) may be categorized into the qualitative-Minor Malformations (MM) like prominent ears, widow’s peak, clinodactyly; and quantitative-Phenogenetic Variants (PV) malformation like short stature, short philtrum, large hands and small feet. The qualitative defects which are considered as true deviations from normal development arising during the period of organogenesis whereas latter are the quantitative defects which are said to arise after the period of organogenesis and are modified during different phases of development.^8^ These anomalies can be considered significant if they could be correlated with the severity of the disease.^9^ Based on the MPAs whether MM or PV, the critical period affecting the brain development can be identified.^8^

In ASD subjects, the incidence of quantitative defects is more than those of the qualitative defects; the inconstant results from different body parts advocate the morphological heterogeneity of autism.^8^ However, there is no study from Pakistan presenting MPAs associated with ASD. This study was conceived to determine the physical morphological characteristics in these subjects.

## METHODS

It was a descriptive cross-sectional study, conducted at the University of Health Sciences (UHS) Lahore Pakistan from September 2016 to October 2020 after obtaining ethical approval from the Ethical Review Committee of the University of Health Sciences Lahore (No. UHS/Education/126-16/499) and Institutional Review Board of Children’s Hospital & Institute of Child Health Lahore (No. CH&ICH01/153/16). The sample size was calculated by a dedicated sample size calculation in the health studies tool by WHO keeping the confidence level equal to 95% and the absolute precision equal to 5%. Using non-probability convenient sampling, 147 subjects aged three years and above with confirmed clinical diagnosis of ASD by Developmental pediatrician, using DSM-V were recruited from CH & ICH Lahore, after written informed consent. All the subjects with known neurological or genetic disorders were excluded from the study. Socio-demographic information, personal and family medical history was obtained on a dedicated data collection form.

### Assessment of Minor Physical Anomalies (MPAs)

Based on the physical examination of 12 body regions (stature; hair growth pattern; ear structure, size and placement; nose size; face size and structure; philtrum; mouth and lips; teeth; hand size; fingers and thumbs; nails; and feet structure and size) in an unclothed subject; all the findings were documented in the ADM manual worksheet.^10^ In addition, physical measurements (height, weight, head circumference, ear length, philtrum, hand, finger and foot length) were also taken and were compared with the available standard charts.^11^ ASD subjects were evaluated by scoring MPAs; the body regions were coded as normal (if there were no dysmorphic features) or abnormal (if dysmorphic features were present). The techniques outlined in previous study were adopted.^10^,^11^

The dysmorphology examination was performed in each subject under supervision of one or more parents. Measurements were taken with the help of a transparent ruler and measuring tape to the nearest 0.1cm; these measurements were taken twice, and the average value was recorded into the database. The measurement of less than the 3rd percentile and more than the 97th percentile was defined as abnormal.

### Statistical analysis

Statistical package for social sciences (SPSS) version 25 for windows was used for the analysis of study data after coding. The normality of the data (such as the number of dysmorphologies) was checked by the Shapiro–Wilk test. Data was considered not normally distributed as p-value was less than 0.05; therefore, Median with IQR (Interquartile range) was calculated for quantitative variables (such as age). For categorical variables (such as dysmorphologies) frequencies and percentages were calculated.

## RESULTS

### Sociodemographic Characteristics of ASD Cohort

These are depicted in Table-I:

**Table I:** Distribution of sociodemographic characteristics of the ASD cohort (n=147).

Parameters (Categorical Variables)		ASD Cohort n (%)
Gender	Male	120 (81.6)
	Female	27 (18.4)
Male:Female ratio		4.5:1
Severity	Mild to moderate	118 (80.3)
	Severe	29 (19.7)
Parental consanguinity		53 (36.1)
Other ASD subject/s in family		16 (10.9)
Developmental/ Psychiatric disorders in family		63 (42.9)

*Parameters (Quantitative Variables)*		*ASD Cohort Median (25^th^-75^th^ Quartile)*

Age of subject at examination (Months)		59 (43-91)
Age at diagnosis (Months)		38 (36-48)
Age at first presentation (Months)		32 (24-42)
CARS (Mild to moderate=36; Severe=37 & above)		34 (31-36)

**n**: Number of subjects, **CARS**: Childhood Autism Rating Scale.

### Dysmorphic Characteristics of ASD Cohort

A total of 381 dysmorphic regions were identified in 131 (89.1%) ASD subjects whereas 16 subjects had no dysmorphology at all. Microcephaly was exhibited by 14 (9.5%) subjects, out of which 13 had variable number of dysmorphologies while one had no dysmorphology in other body regions. Out of 131 subjects exhibiting dysmorphologies, there were 108 male and 23 female subjects, with a M:F ratio 4.7:1 whereas microcephaly was observed in 12 male and two female subjects, with a M:F ratio 6:1.

**Fig.1:**
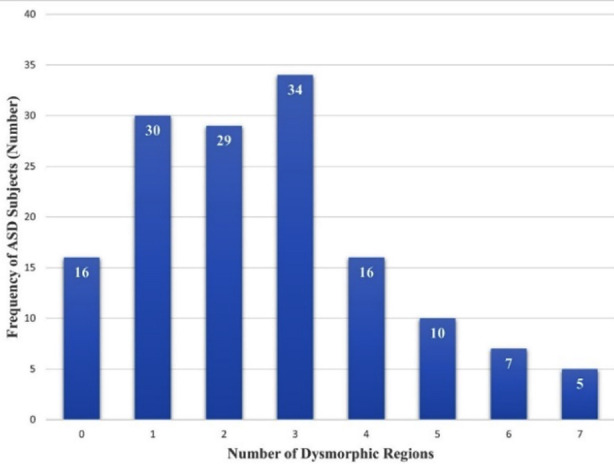
Bar chart showing distribution of number of dysmorphic body regions identified in ASD study cohort (n=147).

**Fig.2:**
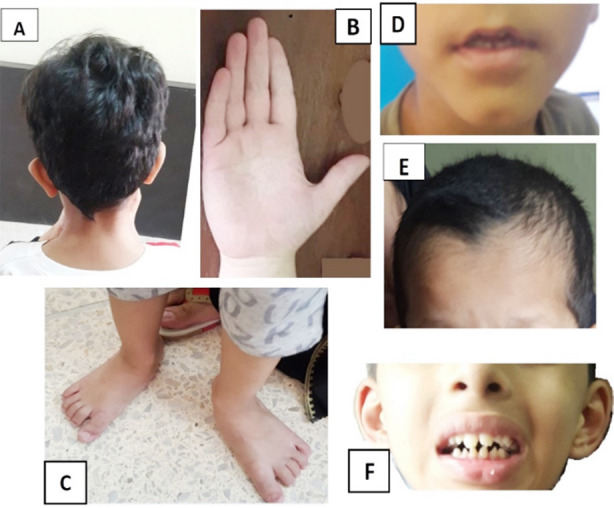
**A)** Low-set & prominent left ear. **B):** Left hand showing clinodactyly of fifth finger. **C)** Short toes bilaterally. **D)** Dimpled chin; simple philtrum; thin upper lip. **E)** Widow’s peak. **F)** Low-set & prominent ears; thick lips; abnormal teeth (printed with permission of parents).

Distribution of dysmorphic regions in the study cohort is shown in Fig-1. **S**ome of the dysmorphologies identified in body regions are shown in Fig-2. There was no statistically significant association between gender and Occipitofrontal circumference (OFC)/ dysmorphic body region in the study cohort.

The highest number of dysmorphologies was noted in the ears, followed by feet and hair growth pattern. The distribution of dysmorphologies in the body region and occipitofrontal circumference in the study cohort is shown in Table-II.

**Table II:** Dysmorphic features in ADM body region and OFC along with their distribution in each body region in the study cohort (n=147).

ADM Body Regions & OFC	ASD subjects n (%)	Dysmorphic features in body regions		Times observed in cohort
Stature	35 (23.8)	Short		24
		Tall		11
Hair Growth Pattern	56 (38.1)	Widow’s peak		14
		Abnormal Anterior Hairline		33
		Abnormal Posterior Hairline		9
		Others^1^		11
Ears	69 (46.9)	Small		5
		Long		33
		Low set		19
		Prominent		19
		Posteriorly rotated		14
		Others^2^		34
Nose	6 (4.1)	Bridge	Broad	3
			Depressed	1
		Bulbous tip		2
		Short		1
Face	21 (14.3)	Facial asymmetry		10
		Dimpled chin		8
		Others^3^		4
Philtrum	21 (14.3)	Short		3
		Deep		4
		Simple		7
		Wide		10
Mouth & Lips	21 (14.3)			
Abnormal mouth	Open mouth look	12		
	Carp-shaped	11		
	Downturned corners	8		
	Asymmetry	7		
	Small	6		
		Wide		2
Abnormal lips	Thin	11		
	Thick	8		
Teeth	19 (12.9)			
Hands	13 (8.8)	Small		5
		Large		8
Fingers & Thumb	42 (28.6)			
Fifth finger	Clinodactyly	25		
	Short	7		
Middle Finger Length	Long	3		
	Short	8		
Nails	1 (0.7)			
Feet & toes	64 (43.5)	Small feet		6
		Large feet		18
		Over-riding toes		39
		Short toes		12
Occipitofrontal Circumference				
Microcephalus	14 (9.5)			
Macrocephalus	05 (3.4)			

In each body region, dysmorphic features were observed in various combinations. Abnormal features observed in only two to three subjects, were categorized as ‘others.’ ^1^. Cowlick/ frontal sweep; abnormal/ unusual hair whorls; coarse, stiff, sparse hair; hair growth on forehead. ^2^. Abnormalities of helix (thick, serpiginous crus of helix), anti-helix (prominent), tragus (bifid), anti-tragus (everted, underdeveloped, prominent), lobule (small), preauricular pit. ^3^. Small face, mid-face retrusion, malar hypoplasia.

## DISCUSSION

Increased incidence of dysmorphic features in subjects with ASD has been reported. By using clinical morphology, the subjects with disrupted structural development during embryogenesis may be delineated. The present study demonstrated dysmorphology assessment, using ADM manual, in a cohort of 147 ASD subjects and also described their sociodemographic, clinical and family characteristics.

Amongst 12 body regions, highest frequency of ear abnormalities (46.9%) was noted; structural anomalies, long, low-set, prominent and posteriorly rotated ears were the most common findings in the current ASD cohort.^10^,^12^ Small ears have also been reported.^13^ However a few reported even higher incidence ranging from 62.6% to 71.5%.^14^, ^15^ The development of external ear occurs from 5^th^ to 22^nd^ week of development when it attains adult structure.^16^ It is, therefore, deviation of normal path of development before 22^nd^ week, leading to anomalies of external ear.

The second most common region with dysmorphologies was feet (43.5%) with higher frequency of over-riding toes and large feet. These results were supported by previous investigators which proposed that minor anomalies are commonly seen in embryologically complex structures like feet.^10^,^14^,^15^,^17^

A high incidence of Hair Growth Pattern (HGP) anomalies (38.1%) was observed in the present ASD cohort; these findings are supported by studies on Indian and Chinese ASD subjects.^14^,^15^ Out of these, anterior hairline abnormalities were most common which is in agreement with the previous.^10^ Brain growth affects the growth of the overlying scalp, and scalp HGP distribution gives valuable information about early intrauterine development (Miles ADM Manual). It may be implied that subtle failure of normal brain growth especially in frontal region of brain during early fetal period (between 10-16 weeks), might be responsible for anomalies of HGP.^18^

We identified 28.6% subjects with anomalies of fingers and thumb; these results were supported by a previous study^15^ whereas a high frequency was reported in other studies.^10^,^14^ Clinodactyly (curved finger) was the most common minor anomaly affecting fifth finger. It had been reported that the middle phalanx of fifth finger is the last digital bone to develop during intrauterine life; hypoplasia of this bone results in clinodactyly.^18^

The frequency of anomalies in face, philtrum, mouth/ lips (21% each) and teeth (19%) in the ASD cohort; however, the anomalies of nose (4.1%) were relatively less. Previously, variable incidence of dysmorphic facial features has been reported. A high frequency of nose anomalies,^10^ anomalies of philtrum, mouth/ lips and teeth,^15^ teeth anomalies,^19^ anomalies of face^14^ has been reported. These facial anomalies may result from minor environmental alterations, genetic composition, or it may be due to natural selection.^20^ It has been proposed that facial involvement is due to dysmorphic development of brain which is reflected onto the head region.^12^

We found 16.3% subjects with short stature. These results were close to those reported in another study in which 19% ASD subjects with short stature in Caucasian population were identified.^10^ Other studies have reported an incidence of 1.3% to 25.5%.^14^,^15^,^21^ This variable incidence of short stature may be due to exclusion and inclusion of syndromic ASD cases in these studies. Short stature observed in subjects showing generalized dysmorphology is an indication that growth failure might occur either prenatally or postnatally.^10^

The anomalies of hands were observed in 8.8% subjects which is similar to that reported in Indian population.^14^ However, there were variable results in other studies; higher incidence in Caucasoid population and slightly lower in Chinese population.^10^,^15^

There was a low tendency towards nail anomalies which was in corroboration with previous results^14^ whereas high frequency was reported in previous studies;^10^,^15^ which indicate compromised development of distal phalanx.^18^

Microcephaly is considered as a biomarker for defining dysmorphology and an important predictor of poor outcome of ASD subjects.^22^ In the present study cohort, microcephaly was observed in 14 (9.5%) subjects with variable number of other dysmorphologies; it is in agreement with previous report.^23^ A variable incidence of microcephaly has been reported in various studies ranging from no microcephalic subject^24^ to as low as 5%^22^ and as high as 40%.^14^ In the microcephalic subjects, different genes might be involved in pathogenesis of ASD.^25^ It may be concluded that the ASD subjects have an insult to early neurodevelopment leading to craniofacial anomalies.^25^

### Strengths and Limitations of the study

The strengths of the study include identification of dysmorphology pattern in ASD in a novel geographic sample set. In addition, a detailed morphological examination of non-syndromic ASD subjects in a prospective way was done, which makes it more reliable as compared to those in which the data is collected from hospital records. However, parents of the subjects could not be examined for dysmorphology due to cultural limitations. For future directions, more efficient way of dysmorphology measure using 3D imaging soft wares is recommended.

## CONCLUSION

High frequency of MPAs has been identified in our ASD cohort both in craniofacial and peripheral body regions which shows morphological heterogeneity of autism. ASD subjects can be clinically classified according to their dysmorphic features and therapeutic plans may be taken accordingly.

### Author’s Contribution:

**AR:** Proposed the study design, conceptualized the project, collected data, prepared the first draft of the manuscript and analyzed the data.

**SM:** Supervised and critically analyzed the project throughout its duration and revised the manuscript critically for important intellectual content.

**SM:** Supervised and critically analyzed the project throughout its duration. All authors checked and approved the final version of the manuscript and are responsible for the integrity of the work.
